# Worm infestations and development of autoimmunity in children – The ABIS study

**DOI:** 10.1371/journal.pone.0173988

**Published:** 2017-03-23

**Authors:** Johnny Ludvigsson, Michael P. Jones, Åshild Faresjö

**Affiliations:** 1 Division of Pediatrics, Dept of Clinical and Experimental Medicine, Linköping University, Linköping, Sweden; 2 Faculty of Human Science, Macquaire University, Sydney, New South Wales, Australia; 3 Department of Medicine and Health, Linköping University, Linköping, Sweden; Baylor College of Medicine, UNITED STATES

## Abstract

Worm infestations influence the immune system and may therefore decrease the risk for autoimmune diseases. The aim of the study was to determine whether children who have developed autoimmune disease were less likely to have had worm infestations in childhood. The ABIS-study is a prospective population-based cohort study of children born in southeast Sweden 1997/99. 17.055 children participated. As of June 2014 116 individuals had developed Type 1 diabetes, 181 celiac disease, and 53 Juvenile Rheumatoid Arthritis. The parents answered questions on worm infestations when the children were 1, 5 and 8 years of age. The ABIS registry was connected to the National Registry of Drug Prescriptions, and national registries for diagnosis of the studied diseases. We found no differences in incidence of worm infestations at 1, 5 or 8 years of age between children who developed autoimmune disease(s) or healthy controls. At 8 years in total 20.0% of the general child population had experienced a worm infestation; children who developed Type 1 diabetes, 21,3%, celiac disease 19,5% and JRA 18,8%. There was no difference in prescriptions of drugs for treatment of worm infestations between those who had and who had not developed Type 1 diabetes, celiac disease, Juvenile Rheumatoid Arthritis. We found no associations indicating that worm infestations in childhood does not play a role in the development of autoimmune diseases in Sweden.

## Introduction

The cause of Type 1 diabetes is unknown. There is a genetic predisposition [1] but several pieces of data strongly suggest that environmental factors must play an important role [2]. Among the different hypothesis increased hygiene has been suspected to play a role [3]. Lack of severe enemies may contribute to deviation of the immune system. Certain viruses may become rare making them severely pathogenic due to a lack of immunity in the population [4]. Gut flora may change the “terror balance” within the immune system, influence maturation of the immune system and cause abnormal immune reactions [5]. In a previous study we were not able to show that certain specific aspects of modern life-style play a role in the development of T1D [6], but this does not rule out the possibility that worm infestations could be important. Worm’s infestations cause a dramatic increase in IgE counts, many-fold higher than usually seen in allergies, however the influence of the immune system seems to prevent clinical manifestations of allergy [7]. The question of whether worm infestation might also decrease the prevalence of autoimmune diseases, such as type I diabetes, is not new [8,9]. Worm infestations cause a pronounced Th2-response in the immune system and to increase regulatory T-cells and prevent the autoimmune reaction of diabetes in NOD-mice [10]. Helminths also lead to increase of IL-10 and TGF-beta counts [11] which may be part of the explanation of the clinical effect in Multiple Sclerosis [12]. With this background, we evaluated whether children who had previously experienced a worm infestation developed Type 1 diabetes, celiac disease or Juvenile Rheumatoid Arthritis (JRA) less often had worm infestations in childhood in a Swedish population.

## Material and methods

The ABIS-study is a prospective population-based cohort study of all children born in southeast Sweden between 1997/99 (N = 21,700) in which 17,055 families (78%) participated. Among these children 116 had developed Type 1 diabetes by 30^th^ of June 2014, 181 had developed celiac disease, and 53 had developed Juvenile Rheumatoid Arthritis (JRA). All three diseases have been validated against national diagnosis registers thanks to the unique 10-digit person number.

The ABIS registry was connected to the National Registry of Drug Prescriptions and we have therefore been able to determine how many children received prescription for drugs against worm infestations. In this present follow-up we have data for 16,420 children.

In addition, when the child was 1 years old the parents were asked to indicate “Has your child had worm infestations” and if answered in the affirmative they were asked to indicate whether this was pinworms, roundworms or other worm infestation. 11,094 questionnaires were available with this information. At 5 years 7,445 and again at 8 years 3,986 of the parents answered questions about whether the child had had a worm infestation, and if so when.

The raw data are registered in the ABIS biobank register, and the right to these data has the founder and project leader Johnny Ludvigsson, who can show where the minimal underlying data set for this study can be found in ABIS biobank register.

### Ethics

The ABIS study has been approved by the Research Ethics Committees at Linköping University (Dnr 99227, Dnr 99321) and Lund University (Dnr LU 83–97), and all parents/guardians gave their informed consent to participate also on behalf of their children. The consent was answered as part of and recorded in the first questionnaire. The ethics committees approved this consent procedure.

### Statistical analysis

All data were stored in a common database and statistically analyzed using the SPSS 23.0 program (SPSS Inc., Chicago, IL, USA). Differences in the proportion of individuals who eventually developed type I diabetes mellitus (T1D), Juvenile Rheumatoid Arthritis (JRA) and coeliac disease were compared across groups who had and did not have worm infestations at age 1 year, 5 years and 8 years using the Pearson Chi-Square test. A number of potentially confounding variables were considered, gender of the child, type of locality the child was born into, medications that might mask the infection, low educational attainment (year 9 or lower) of mother and of father. These variables were compared between those who eventually developed each outcome (T1D, JRA and coeliac disease) and those who did not to establish one of the essential criteria for confounding. The association between worm infestation and outcome was re-assessed controlling for potentially confounding variables using unconditional logistic regression. Adjusted associations between worm infestation and outcomes are reported as odds ratios with 95% confidence intervals and p-values.

## Results and discussion

At one year of age 11,094 questionnaires available which had been completed by the parents. Those children who were reported as having have had a worm infestation divided into, 94% pinworms, 5% roundworms and 4% other worm infection, including a small number with multiple worm types. There was no difference between boys (0.8%) and girls (0.9%, p = 0.73) or whether the children were brought up in town or countryside (Smallest location 1.5% and largest location 1.3%, p = 0.846) and we found no association with parental education (mothers p = 0.65 and fathers p = 0.51) None of the children who later developed Type 1 diabetes had recorded a worm infestation before 1 year of age. On the other hand, only one and two children developed celiac disease and JRA, respectively. At 5 years we had questionnaire data from n = 7,445 children, of whom in total 14.5% had experienced a worm infestation. The corresponding figures for children who developed Type 1 diabetes were also 14.5%, for celiac disease 12.9% and for JRA 9.5%.

At 8 years n = 3,936 children provided questionnaire data, of whom 20.0% (n = 786) had recorded a worm infestation. The corresponding figures for children who developed type 1 diabetes were 21.3%, celiac disease 19.5% and JRA 19.0%, see S1 Table.

Very few children 3.0% (n = 476) of the total ABIS cohort (n = 16.423 had ever been prescribed drugs for worm infestation. In those who had developed Type 1 diabetes worm medication had been prescribed for 1.7% compared with 2.9% without diabetes, (p = 0.45), JRA 0% compared with 2.9% without JRA (p = 0.21), while there was a tendency that somewhat more of the children with celiac disease had been prescribed drugs 5.0% compared with 2.9% without celiac disease, (p = 0.09). Prescription of drugs against worm infestation in different ages is shown in S2 Table.

Only n = 39 children reported past worm infestation at both 1 and 8 years of age and 25/39 also reported past worm infestation at 5 years of age. To date none of them have developed type I diabetes, celiac disease or JRA. Of these individuals 3/39 (7.6%) had been prescribed drugs for worm infestation. Of all potentially confounding variables considered with respect to all outcomes only gender of the child was associated with JRA and gender of the child and low paternal education were associated with coeliac disease. Association between worm infestation and outcome adjusted for potential confounding were tested. Only worm infestation at age 1 year was associated with development of RA after controlling for potentially confounding variables (S3 Table).

No clear evidence was found that prior worm infestation is a risk or protective factor for either RA or coeliac disease at any age with the exception of RA at age one year. In one year olds the odds of RA were substantially elevated in children who had experienced worm infestation (odds ratio 4.66, S3 Table. In contrast, at age 5 prior worm infestation demonstrated some evidence as a protective factor against RA (odds ratio 0.54) but this failed to reach statistical significance (p = 0.4) due to the small number of JRA cases and consequent low statistical power.

Worm infestations may influence the immune system and cause a pronounced Th2 reaction with very pronounced production of IgE (7). It has also been shown that worm infestations increase regulatory T-cells and prevent the autoimmune form of diabetes in NOD-mice (10). Helminths also lead to increase of IL-10 and TGF-beta [11]. It has therefore been suggested that worm infestations, as part of the hygiene hypothesis, might decrease the risk of autoimmune diseases such as Type I diabetes [8,9]. However, our lack of association between worm infestations and Typ1 diabetes may have many explanations. Helminth infections are associated with many different immunomodulatory mechanisms that affect the host immune response. This modulation of host immunity has both advantageous and disadvantageous consequences. Thus the host can benefit from reduced autoimmune, and inflammatory, as well as allergic, reactions. However, helminth infection can also have negative effects and eg increase susceptibility to infection. The possible mechanisms are very well described in a review by Maizels RM and McSorley HJ [13]. Furthermore, worm manifestations may be less effective at distant autoimmune sites or intensity/duration of worm infestation may be determinative in whether these are immunomodulatory, and in this study we do not know how heavy the worm burden has been. This might be another explanation to our lack of association. Finally, no single study can yield a globally generalizable and chronologically-invariant finding, as Type 1 diabetes is a heterogeneous disease and different environmental factors may play a role in different parts of the world and in different times and periods of time. Thus there are certainly major differences in hygiene factors between the Swedish and some other populations in the world. That said, in this Swedish study we find no association supporting the hygiene hypothesis with respect to worm infestation and several autoimmune diseases.

The strength of this study is that, thanks to the national diagnosis registries and associated unique personal identification number, we know that we have identified all individuals in the entire population who have developed Type 1 diabetes, Juvenile rheumatoid arthritis and celiac disease in this cohort. Regarding prescribed drugs for worm infestations, we can obtain exact due to the capacity to link the ABIS data with the national registry for drug prescriptions. However, as most parents either do not at all treat, buy medications that do not require a prescription by physicians, registry data is unlikely to be complete. However, in the ABIS study we have prospective registration of what parents regard as worm infestations, something most parents are quite aware of. This shows that many children have most probably had worm infestations even though they have not been prescribed drugs by physicians. This recording by the parents was done without any bias towards development of a certain diseases such as allergy, diabetes etc as ABIS has been following a broad general population, not restricted to a special disease or to special heredity or certain HLA-types. Some potentially confounding variables were also considered with respect to all outcomes. The analysis showed that only gender of the child was associated with JRA. Further, gender of the child and low paternal education were associated with coeliac disease.

A weakness is the drop-out rate for questionnaires. However, we know that the population responding is quite representative of the general Swedish population except for a slightly lower frequency of parents with “other” ethnic origin [14,15], and therefore we believe that our results are reliable and representative. For the drug prescriptions there are, by definition, no drop outs as the national register of drug prescriptions covers all children in Sweden.

### Conclusions

Although increased hygiene and change in infectious spectrum may influence both the immune system/balance and possibly the development of both allergies and autoimmune disease, in this prospective cohort study we found no associations indicating that worm infestations in childhood play any role in the development of autoimmune diseases such as Type 1 diabetes in Sweden. However, we cannot exclude the possibility that helminths may play a role for prevention of autoimmune diseases in other parts of the world, where perhaps the children have more chronic helminth infections as well as other infections that counteract the imbalance allowing autoimmune reactions.

## Supporting information

S1 Table Comparison of percentage of each outcome across individuals who did and did not have worm infestations at ages 1, 5, 8 and both 1 and 8. (DOCX)

S2 Table Worm infestation and prescription of drugs. (DOCX)

S3 Table Adjusted associations between worm infestation and outcomes. (DOCX)
