# Supplementary material for: Worm infestations and development of autoimmunity in children – The ABIS study
Source: PLoS One. 2017 Mar 23;12(3):e0173988. doi: 10.1371/journal.pone.0173988 (PMC5363823; doi:10.1371/journal.pone.0173988)
Supplement: S1 Table — (DOCX) [file pone.0173988.s001.docx]

PONE-D-16-22183R2

Table 1  **Comparison of percentage of each outcome across individuals who did and did not have worm infestations at ages 1, 5, 8 and both 1 and 8.**

| **Worm**  **infestation** | **Diabetes n=116** | | | | | **Coeliac n=181** | | | | | **Juvenil Rheumatoid Arthritis**  **n=53** | | | | |
| --- | --- | --- | --- | --- | --- | --- | --- | --- | --- | --- | --- | --- | --- | --- | --- |
| **Age** | **Yes**  **n** | **%** | **No**  **n** | **%** | **p-value** | **Yes**  **n** | **%** | **No**  **n** | **%** | **p-value** | **Yes**  **n** | **%** | **No**  **n** | **%** | **p-value** |
| **1** | 0 | 0.0 | 137 | 0.8 | 0.32 | 1 | 0.6 | 136 | 0.8 | 0.72 | 2 | 3.8 | 135 | 0.8 | 0.02 |
| **5** | 9 | 14.5 | 1075 | 15.3 | 0.86 | 9 | 13.0 | 1075 | 15.3 | 0.56 | 2 | 9.5 | 1082 | 15.3 | 0.46 |
| **8** | 10 | 21.3 | 776 | 20.0 | 0.82 | 8 | 19.5 | 778 | 20.0 | 0.94 | 3 | 19.0 | 783 | 20.0 | 0.90 |
| **1 and 8** | 0 | 0.0 | 39 | 0.2 | 0.59 | 0 | 0.0 | 39 | 0.2 | 0.51 | 0 | 0.0 | 39 | 0.2 | 0.72 |
