# Supplementary material for: Worm infestations and development of autoimmunity in children – The ABIS study
Source: PLoS One. 2017 Mar 23;12(3):e0173988. doi: 10.1371/journal.pone.0173988 (PMC5363823; doi:10.1371/journal.pone.0173988)
Supplement: S2 Table — (DOCX) [file pone.0173988.s002.docx]

PONE-D-16-22183R2

Table 2: **Worm infestation and prescription of drugs.**

|  | **Children**  **1 year**  **(N = 11.094)** | **Children**  **5 year**  **(N = 7.445)** | **Children**  **8 year**  **(N= 3.986)** |
| --- | --- | --- | --- |
|  | **n %** | **n %** | **n %** |
| **Children with Worm infestation** | 137 1.2 | 1084 14.5 | 786 20.0 |
| **Prescription of drugs against worm infestation for these affected children** | 7 5.1 | 103 9.5 | 84 10.7 |
