# Supplementary material for: Worm infestations and development of autoimmunity in children – The ABIS study
Source: PLoS One. 2017 Mar 23;12(3):e0173988. doi: 10.1371/journal.pone.0173988 (PMC5363823; doi:10.1371/journal.pone.0173988)
Supplement: S3 Table — (DOCX) [file pone.0173988.s003.docx]

PONE-D-16-22183R2

Table 3: **Adjusted associations between worm infestation and outcomes.**

|  | | | **95% Confidence interval** | |  |
| --- | --- | --- | --- | --- | --- |
| **Age** | **Outcome** | **OR** | **Lower** | **Upper** | **p-value** |
| **1** | **RA** | 4.66 | 1.12 | 19.36 | 0.03 |
| **1** | **Coeliac** | 0.65 | 0.09 | 4.65 | 0.67 |
| **5** | **RA** | 0.54 | 0.13 | 2.33 | 0.4 |
| **5** | **Coeliac** | 0.79 | 0.39 | 1.60 | 0.5 |
| **8** | **RA** | 0.90 | 0.26 | 3.16 | 0.9 |
| **8** | **Coeliac** | 0.95 | 0.44 | 2.06 | 0.9 |
